# Application of patient-derived organotypic tumor spheroids to guide combination immunotherapy for advanced HCC: a case report

**DOI:** 10.3389/fonc.2026.1744326

**Published:** 2026-06-02

**Authors:** Fu-Yi Wang, Li-Long Zhu, Cheng Ji, Jie Deng, Lu Han, Fei Song, Zhong Chen

**Affiliations:** 1 Department of Hepatobiliary Surgery, Affiliated Hospital of Nantong University, Medical School of Nantong University, Nantong, China; 2 Medical Service Center, Jiangsu Vocational College of Medicine, Yancheng, Jiangsu, China

**Keywords:** combination immunotherapy, drug sensitivity testing, hepatocellular carcinoma (HCC), patient-derived organotypic tumor spheroids (PDOTS), personalized medicine, tumor microenvironment

## Abstract

**Background:**

Predicting response to combination immunotherapy in advanced hepatocellular carcinoma (HCC) remains a challenge. The patient-derived organotypic tumor spheroid (PDOTS) model, which may preserve aspects of the tumor immune microenvironment, represents a potentially promising tool for personalized therapy guidance, but its clinical utility in HCC requires further validation.

**Case presentation:**

A PDOTS model was generated from a biopsy of a 64-year-old man with advanced HCC (massive tumor with portal vein thrombus). Drug sensitivity testing(DST) in the model suggested sensitivity (Organoid Killing Index=50%) to the combination of Lenvatinib and Tislelizumab, but not to either agent alone. Based in part on this result, the patient received this combination regimen. After 6 cycles, imaging showed a reduction in tumor size, and serum biomarkers (AFP, PIVKA-II) normalized, achieving a partial response that was sustained for 14 cycles.

**Discussion:**

The observed clinical response was concordant with the PDOTS prediction. This case suggests that the PDOTS model may partially recapitulate tumor-immune interactions in HCC, addressing a key limitation of traditional models. Its ability to identify a potentially responsive combination regimen highlights its possible utility in guiding treatment decisions. The main limitation is the single-case nature of this report.

**Conclusion:**

This study provides preliminary, hypothesis-generating evidence that the PDOTS model may be associated with treatment response to Lenvatinib plus Tislelizumab in advanced HCC, offering a potential platform for personalized immunotherapy selection. Further prospective studies are warranted to validate its predictive value.

## Introduction

Primary hepatocellular carcinoma (HCC) is a leading cause of cancer-related death worldwide, with a particularly severe disease burden in China (accounting for 55% of global HCC-related deaths) (1, 2). Due to the lack of specific symptoms in early stages, over 60% of patients are diagnosed at an intermediate or advanced stage (BCLC B/C or CNLC IIb/III), where traditional surgical resection is often not feasible (3, 4). Systemic drug therapy, including targeted therapy and immunotherapy, thus becomes the key treatment modality for unresectable HCC (5, 6). Recent studies have confirmed that immunotherapy combined with anti-angiogenic therapy significantly improves the objective response rate (ORR) in advanced HCC (6, 7). However, even combination regimens (e.g., Atezolizumab plus Bevacizumab) achieve ORRs of only 30-35% (8), still below 40%, with significant individual heterogeneity. Therefore, there is an urgent need for individualized predictive models capable of better anticipating treatment response.

Traditional drug sensitivity models, such as patient-derived xenograft (PDX) models, are inadequate for predicting immunotherapy response because they cannot effectively simulate the tumor immune microenvironment or lack a functional human immune system (9). Addressing this bottleneck, the Patient-Derived Organotypic Tumor Spheroids (PDOTS) model developed by R.W. Jenkins et al. in 2018 has been proposed as a significant breakthrough (10). The core innovation of this model lies in the co-culture of fresh patient tumor tissue with autologous tumor-infiltrating lymphocytes (TILs) within a 3D microfluidic chip, thereby potentially preserving the primary tumor’s immune microenvironment, extracellular matrix structure, and T-cell functional activity. This provides a more physiologically relevant platform for the *in vitro* evaluation of immune checkpoint inhibitor efficacy (11–13). This model was first successfully applied and validated in non-small cell lung cancer (NSCLC), where it’s *in vitro* sensitivity to programmed death receptor-1 (PD-1) blockade was reported to correlated with clinical patient response (predictive accuracy reached 88%), offering a potentially useful predictive tool for personalized immunotherapy decisions in patients with solid tumors, including HCC (10, 12).

This article reports a case of a patient with stage C massive HCC accompanied by portal vein tumor thrombus(PVTT). A PDOTS model was successfully constructed using the patient’s biopsy sample and subjected to drug sensitivity testing. The results suggested potential sensitivity to the combination of Lenvatinib and Tislelizumab. Based on these findings, the patient received the combination therapy. Follow-up evaluation after 6 treatment cycles showed significant tumor shrinkage on imaging, achieving a partial response (PR) (14). The clinical efficacy was concordant with the drug sensitivity prediction from the PDOTS model, providing a preliminary proof-of-concept observation supporting the feasibility of using the PDOTS model in guiding individualized HCC treatment, while further validation in larger cohorts remains necessary.

## Case report

**Figure 1:**
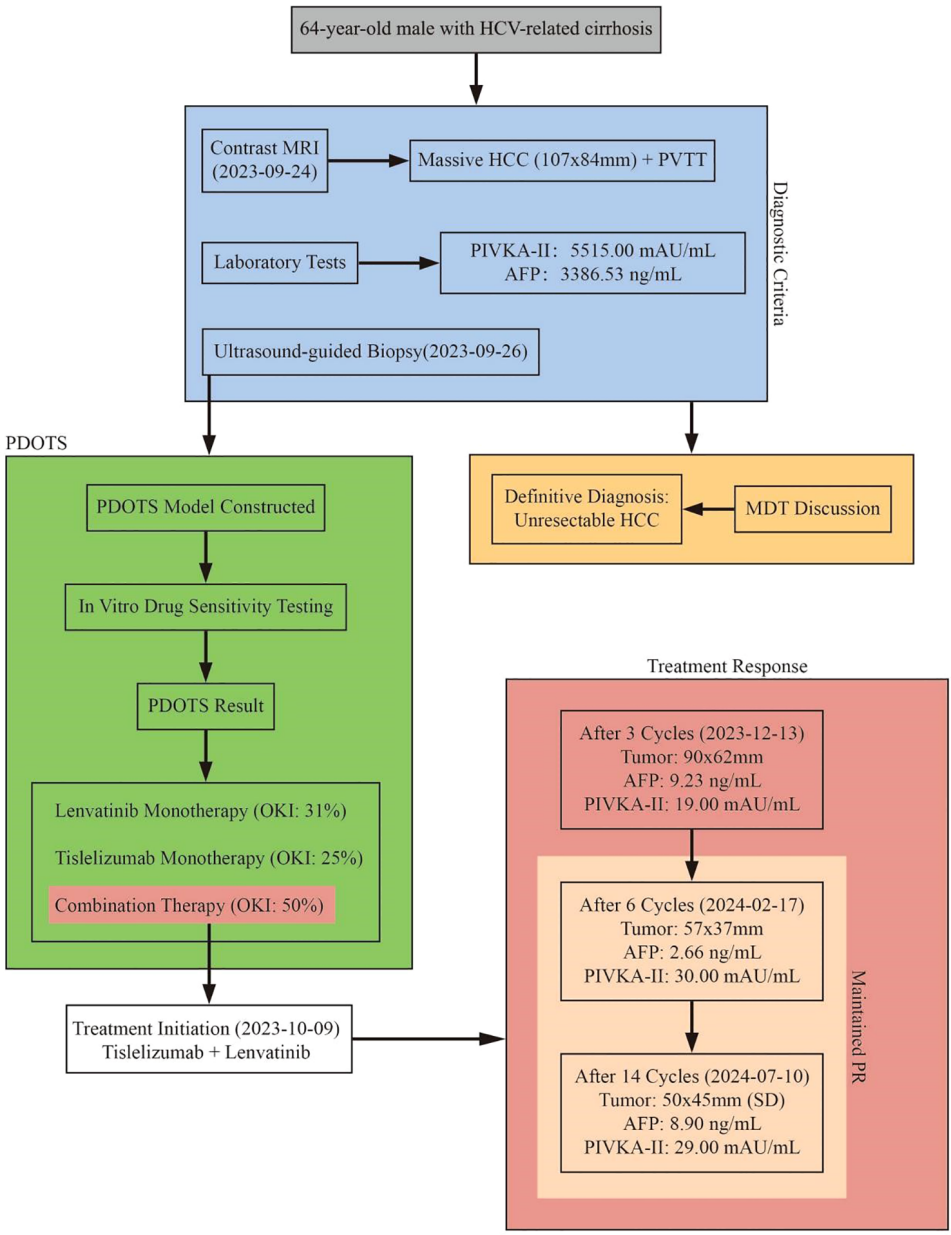
Flowchart of the patient’s diagnostic evaluation, PDOTS construction, treatment decision-making, and clinical follow-up. This figure summarizes the complete clinical workflow from initial diagnosis to longitudinal treatment follow-up in a patient with advanced hepatocellular carcinoma (HCC). Key steps include radiologic examination, histopathological confirmation by liver biopsy, construction of the patient-derived organotypic tumor spheroid (PDOTS) model, ex vivo drug sensitivity testing (DST), selection of the Lenvatinib plus Tislelizumab regimen based in part on PDOTS findings, serial imaging evaluation according to modified Response Evaluation Criteria in Solid Tumors (mRECIST), and dynamic monitoring of serum alpha-fetoprotein (AFP) and protein induced by vitamin K absence or antagonist-II (PIVKA-II) levels.

**Figure 2:**
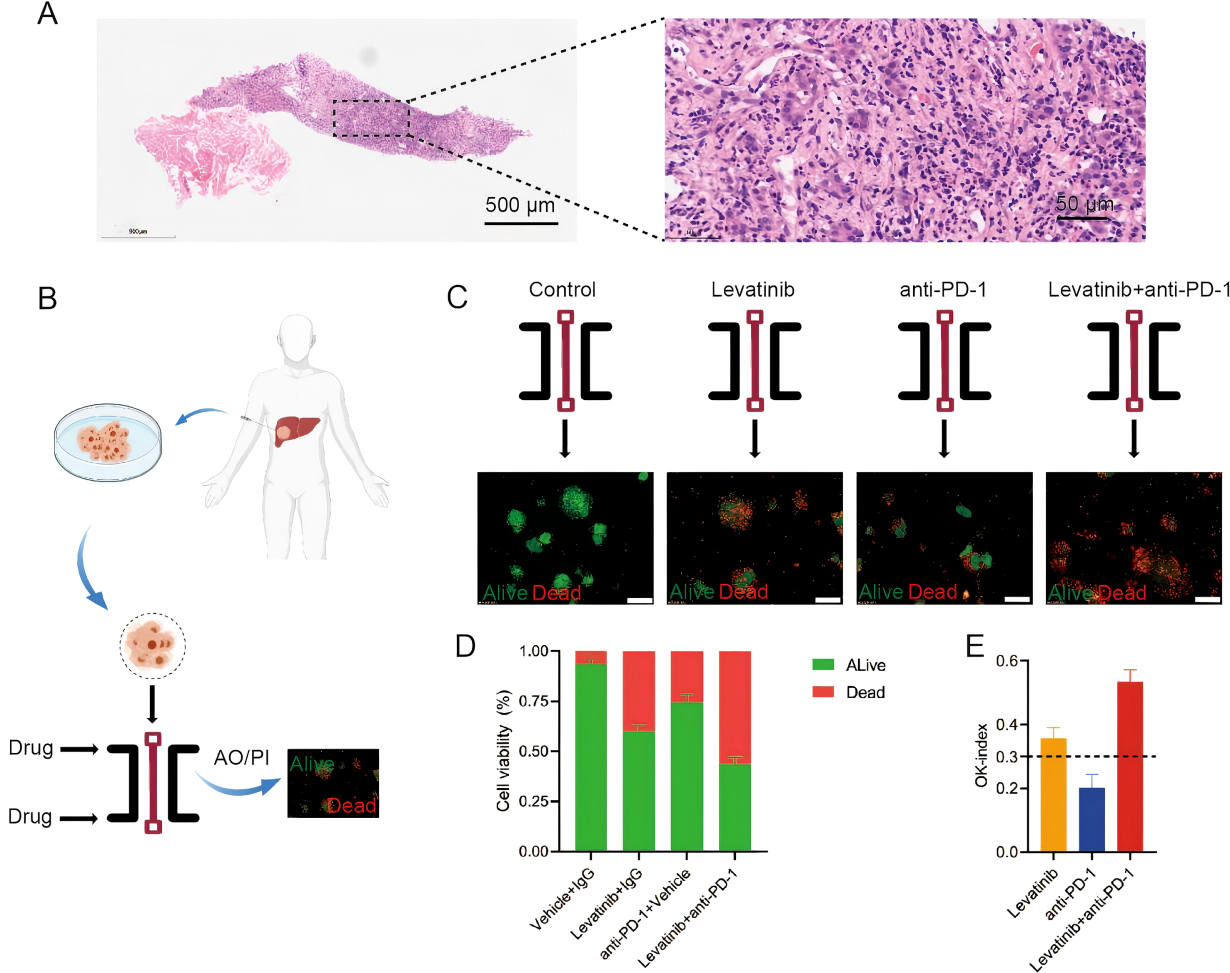
PDOTS model demonstrates highest relative sensitivity to the combination of Tislelizumab and Lenvatinib. **(A)** Hematoxylin and eosin (H&E) staining of the liver biopsy specimen showing hepatocellular atypia consistent with hepatocellular carcinoma (HCC) (scale bar: 100 μm). **(B)** Schematic illustration of the patient-derived organotypic tumor spheroid (PDOTS) model construction workflow. Fresh biopsy-derived tumor tissue was mechanically fragmented, embedded in type I collagen, cultured within a 3D microfluidic chip system, followed by ex vivo drug treatment and acridine orange/propidium iodide (AO/PI) fluorescence staining analysis. **(C)** Representative fluorescence images of AO/PI-stained PDOTS after 5 days of treatment under different experimental conditions, including control, Tislelizumab monotherapy, Lenvatinib monotherapy, and combination treatment groups. Green fluorescence indicates viable cells, while red fluorescence indicates dead cells (scale bar: 200 μm). **(D)** Quantitative analysis of relative cell viability in each treatment group based on ImageJ fluorescence intensity analysis. Each condition was performed with at least three technical replicates. **(E)** Organoid Killing Index (OKI)-based drug sensitivity assessment. Based on the pre-defined sensitivity criterion (OKI > 40% considered sensitive), the combination regimen demonstrated the highest relative sensitivity (OKI = 50%) compared with monotherapy groups.

A 64-year-old male patient with a 10-year history of chronic hepatitis C-related cirrhosis was incidentally found to have a space-occupying lesion in the right hepatic lobe during routine follow-up imaging at a local hospital. For definitive diagnosis and treatment planning, the patient was admitted for systematic evaluation. **Figure 1** outlines the complete diagnostic and therapeutic process in a flowchart. Contrast-enhanced upper abdominal CT revealed a large mass in the right hepatic lobe (107mm × 84mm) with a filling defect in the right portal vein consistent with tumor thrombus. Additionally, multiple enlarged lymph nodes in the hepatogastric space and retroperitoneum, an irregular liver morphology with heterogeneous density (consistent with cirrhosis), splenomegaly, gastric varices (suggesting portal hypertension), and ascites in the abdominal and pelvic cavities were observed. Laboratory tests showed significantly elevated serum tumor markers: alpha-fetoprotein (AFP) at 3386.53 ng/mL and protein induced by vitamin K absence or antagonist-II (PIVKA-II/DCP) at 5515.00 mAU/mL. These imaging features (massive HCC with portal vein tumor thrombus) combined with markedly elevated tumor markers were consistent with hepatocellular carcinoma (HCC). To obtain histopathological confirmation, the patient underwent an ultrasound-guided percutaneous liver biopsy on October 1, 2023. Histological examination (**Figure 2A**) showed hepatocellular atypia. Immunohistochemical staining was positive for the hepatocyte marker (Hepatocyte, diffusely strong positive), Cytokeratin 18 (CK18, positive), Glypican-3 (focally positive), and the Ki-67 proliferation index was approximately 40% (indicating high proliferative activity). Based on the histomorphology and characteristic immunohistochemistry, the diagnosis of hepatocellular carcinoma (HCC) was confirmed. In summary, the diagnosis was based on: (1) Underlying chronic liver disease (HCV cirrhosis); (2) Typical imaging features (massive HCC with first-order branch PVTT, signs of cirrhotic portal hypertension); (3) Significantly elevated serum AFP and PIVKA-II levels; (4) Histopathological confirmation (biopsy morphology and supportive immunohistochemistry). According to the China Liver Cancer (CNLC) staging criteria from the “Guidelines for the Diagnosis and Treatment of Primary Liver Cancer (2024 Edition)”, the clinical stage was determined as stage C due to the maximum tumor diameter >10 cm and involvement of the first-order branch of the portal vein. Consistently, based on the Barcelona Clinic Liver Cancer (BCLC) staging system, the patient was classified as stage C due to the presence of portal vein tumor thrombus, indicating advanced disease and lack of surgical eligibility.

To formulate an individualized treatment plan, a microfluidic PDOTS model was successfully constructed using the patient’s biopsy tumor tissue (**Figure 2B**) (detailed construction process is described in the Methods section). After culture in a 3D microfluidic collagen environment (13, 15), the model was treated with Tislelizumab (anti-PD-1 antibody), Lenvatinib (multi-target tyrosine kinase inhibitor) as monotherapies, or their combination. Following staining, the PDOTS were observed under a fluorescence microscope to distinguish live (green) from dead (red) cells (**Figure 2C**). Cell viability was quantified using ImageJ software by analyzing the balance value of red and green fluorescence intensities (**Figure 2D**). The DST results showed OKIs of 31% for the Lenvatinib monotherapy group, 25% for the Tislelizumab monotherapy group, and 50% for the combination therapy group. Based on the pre-defined sensitivity criterion (OKI > 40% considered sensitive) (13, 15), the combination regimen showed the highest relative sensitivity (OKI = 50%) and was therefore considered as a potential treatment option. (**Figure 2E**) (Organoid Killing Index, OKI = 1 - Relative Viability Rate; Relative Viability Rate defined as live cell signal in treatment group/live cell signal in control group).

**Figure 3:**
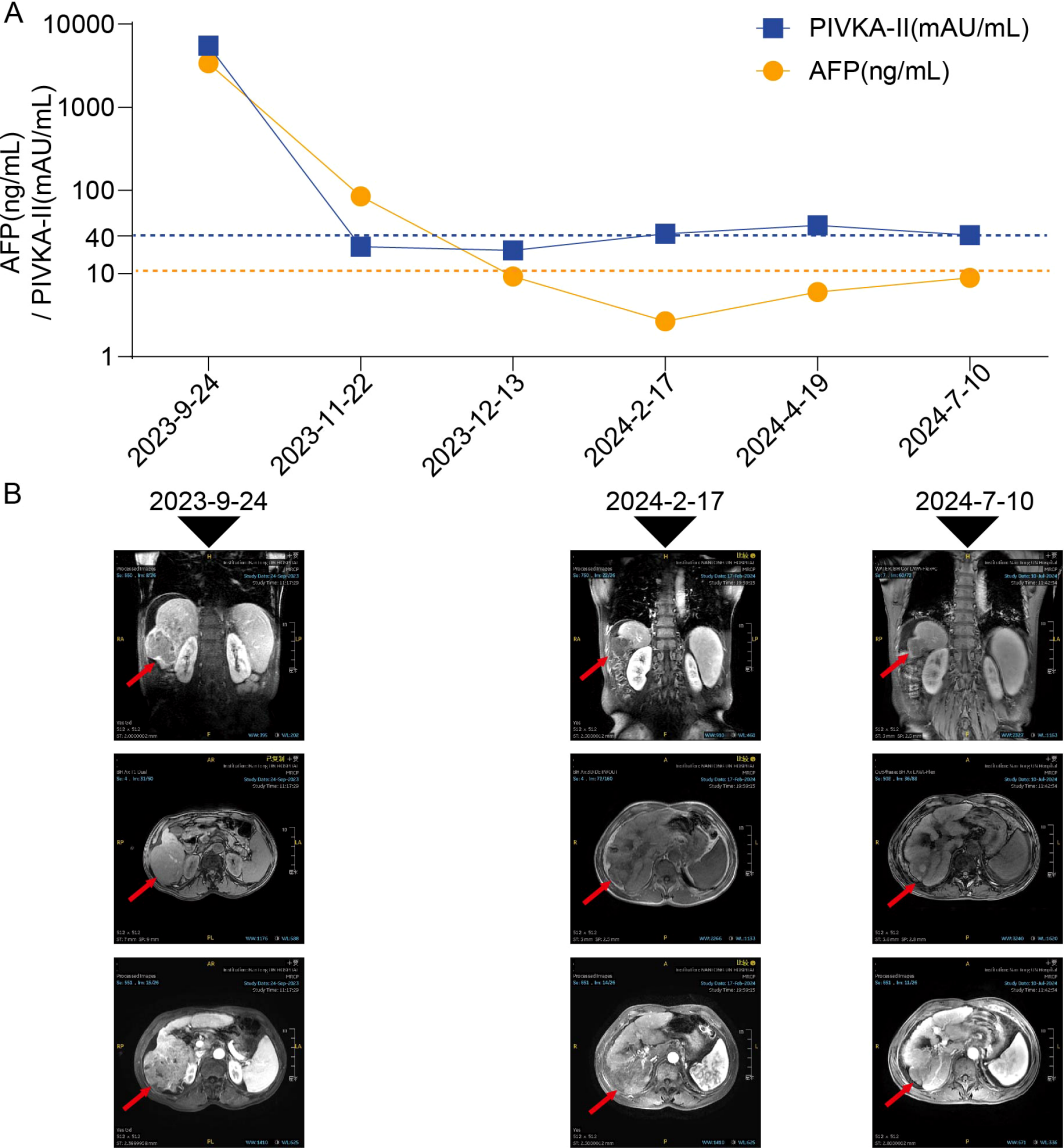
Longitudinal clinical follow-up after administration of the treatment regimen supported by the PDOTS model. **(A)** Dynamic changes in serum alpha-fetoprotein (AFP) and protein induced by vitamin K absence or antagonist-II (PIVKA-II) levels during treatment follow-up. Both biomarkers showed a marked decline after initiation of Lenvatinib plus Tislelizumab therapy and subsequently remained at low levels during continued treatment. **(B)** Serial contrast-enhanced magnetic resonance imaging (MRI) examinations obtained at baseline (pre-treatment), after 3 treatment cycles, and after 6 treatment cycles. Imaging demonstrated progressive shrinkage of the primary lesion in the right hepatic lobe together with reduction of portal vein tumor thrombus (PVTT). Red arrows indicate tumor borders and regions of interest. Radiologic response was evaluated according to modified Response Evaluation Criteria in Solid Tumors (mRECIST), and partial response (PR) was achieved after 6 cycles of treatment.

Based on the PDOTS model prediction, the patient began treatment on October 9, 2023, with Lenvatinib (8 mg, orally, once daily) combined with Tislelizumab (200 mg, intravenous infusion, every 3 weeks). Treatment response and follow-up are summarized (**Figure 3**):

• After 3 cycles of treatment (December 13, 2023): Follow-up contrast-enhanced CT showed a reduction of the right hepatic lobe tumor to 90mm × 62mm.
• After 6 cycles of treatment (February 17, 2024): Follow-up contrast-enhanced upper abdominal MRI showed further reduction of the tumor to 57mm × 37mm, with reduction in the right portal vein tumor thrombus; serum AFP decreased to 2.66 ng/mL, and PIVKA-II decreased to 30.00 mAU/mL. Efficacy was evaluated as partial response (PR) according to mRECIST criteria, based on the sum of diameters of viable enhancing tissue in the target lesion (the right hepatic lobe mass) and the portal vein tumor thrombus. The non-enhancing necrotic component was excluded from measurement.
• Treatment was continued with follow-up until the 14th cycle (July 10, 2024). Repeat contrast-enhanced upper abdominal MRI showed stable primary lesion (50mm × 45mm), with minimal change from the previous scan, indicating stable disease; the patient maintained PR status for 14 cycles from the start of treatment.

## Methods

### Patient and sample

We collected samples from the tumor biopsy tissue of this patient with unresectable hepatocellular carcinoma to construct a microfluidic PDOTS model and test sensitivity to commonly used targeted and immunotherapeutic drugs. Given the limited tissue quantity obtained from percutaneous core needle biopsy, a modified sampling strategy adapted from the previously reported “Five-Point Clock Method” was employed to partially address potential intratumoral spatial heterogeneity17. Specifically, two biopsy cores were obtained along different spatial axes of the tumor lesion under ultrasound guidance. Each biopsy core was longitudinally divided into multiple small fragments and subsequently pooled for downstream PDOTS establishment. Unlike surgically resected specimens, comprehensive multi-regional sampling using the conventional Five-Point Clock Method was not feasible in this biopsy-based setting due to limited tissue availability. Tumor response assessment followed the mRECIST criteria (16). Serum AFP levels were measured using a chemiluminescent immunoassay system (Abbott Laboratories, Chicago, IL, USA). Serum PIVKA-II levels were determined using a chemiluminescent enzyme immunoassay kit (Fujirebio Inc., Tokyo, Japan) according to the manufacturer’s instructions. Contrast-enhanced MRI examinations were performed using a 3.0-T scanner (MAGNETOM Skyra, Siemens Healthineers, Erlangen, Germany). Gadoxetic acid disodium was used as the contrast agent at a dose of 0.1 mL/kg. Standard liver imaging sequences, including T1-weighted imaging, T2-weighted imaging, diffusion-weighted imaging, and dynamic contrast-enhanced phases, were acquired according to institutional protocols. This study was reviewed and approved by the Research Ethics Committee of the Affiliated Hospital of Nantong University (Approval No. 2022-L062). All procedures performed in this study were in accordance with the ethical standards of this institutional committee and with the 1964 Helsinki Declaration and its later amendments. Written informed consent was obtained from the patient for the publication of this case report and any accompanying images and data.

### Construction of a 3D culture system for patient-derived organotypic tumor spheroids using a microfluidic chip

The fresh patient tumor specimen obtained using the Five-Point Clock Method described above was placed in ice-cold DMEM medium and minced on ice in a sterile environment within a 6-cm culture dish. PDOTS, with diameters ranging from 40 to 100 μm (the S2 fraction), were generated ex vivo following previously established protocols (17). Specifically, the 40-100 μm S2 fraction was taken for ex vivo culture. The S2 fraction was resuspended in Type I rat tail collagen solution (Corning, Cat. No. 354236; USA), and then the spheroid-collagen mixture was injected into the gel center region of the 3D microfluidic culture device (AIM Biotech, Cat. No. DAX-1; Singapore). After incubation at 37°C for 30 minutes, the collagen hydrogel containing PDOTS was hydrated with medium containing specific treatment conditions (13). The specific culture medium consisted of Advanced DMEM/F12 (Gibco, 12634-010) supplemented with 10% fetal bovine serum (FBS) (Gibco, 10270-106), recombinant human IL-2 (500 U/mL, PeproTech, 200-02-100), and ImmunoCult™ Human CD3/CD28 T Cell Activator (STEMCELL, 10971). PDOTS were co-cultured for 7 days (17). Finally, the PDOTS were treated with Tislelizumab (10 μg/mL), Lenvatinib (10 μM), or the combination regimen under standard culture conditions. After 5 days of drug exposure, the culture medium was carefully removed, and the PDOTS were stained with 10 μL AO/PI staining solution (Nexcelom Bioscience, Lawrence, MA, USA) in the dark at 4°C for 5 minutes. Live (green) and dead (red) cells were subsequently visualized using fluorescence microscopy. Cell viability was quantified using ImageJ software by analyzing the balance value of red and green fluorescence intensities. Each experimental condition was performed with at least three technical replicates (17).The Organoid Killing Index (OKI) was derived as follows:


$$Alive\;=\;AG/\left(AG\;+\;AR\right)$$

ΔAlive = Alive_NC − Alive_T

$$OKI\;=\;\left(\Delta Alive/Alive\_NC\right)\;\times \;100\%$$

Where AG and AR represent the area of green fluorescence and red fluorescence, respectively. Alive_NC and Alive_T represent the cell viability of the negative control group and the drug-treated group, respectively (13, 15). As the OKI is calculated relative to the untreated control, it inherently normalizes for baseline, non-specific toxicity Based on the pre-defined sensitivity criterion established in our prior study, an OKI > 40% was considered sensitive (17).

## Discussion

This case report describes the use of the Patient-derived organotypic tumor spheroids (PDOTS) model (18) to inform personalized treatment decision-making for a patient with BCLC stage C advanced hepatocellular carcinoma (HCC). The patient was diagnosed with massive HCC accompanied by portal vein tumor thrombus, which was considered unresectable. Using the biopsy tissue, a PDOTS model was constructed. Within this 3D culture that may preserve key components of autologous tumor-infiltrating lymphocytes (TILs) and the tumor microenvironment (TME), *in vitro* drug sensitivity testing was performed for Lenvatinib, Tislelizumab monotherapies, and their combination. The DST suggested relative sensitivity to the Lenvatinib plus Tislelizumab regimen (OKI index reached 50%). After clinical implementation of this regimen, the patient’s tumor decreased in size (from 107mm×84mm to 50mm×45mm), serum AFP and PIVKA-II levels nearly normalized, and a partial response (PR) was maintained for 14 cycles. The clinical response was concordant with the sensitivity findings from the PDOTS model, providing a preliminary proof-of-concept observation rather than definitive predictive validation. This case suggests the potential feasibility of PDOTS as a personalized treatment decision-support tool in solid tumors, particularly in HCC where response to immunotherapy is highly heterogeneous.

Compared to traditional models, this study highlights the potential advantages of PDOTS in exploring immunotherapy response. Organoid (PDO) or patient-derived xenograft (PDX) models are inadequate for assessing immune checkpoint inhibitor efficacy due to the lack of functional immune cells (11, 18). For instance, the HCC organoids established by Broutier et al. in 2017 could simulate tumor epithelial characteristics but failed to incorporate the complete TME, including immune cells (19). PDX models, besides lacking a relatively intact immune microenvironment, also involve considerable time and economic costs. Although recent studies have attempted co-culturing peripheral blood lymphocytes (PBLs) with PDOs (e.g., Neal et al., Nature Medicine 2018) (20), the composition and functional status of immune cells in the reconstituted TME still differ significantly from*in situ* TILs. The core advantage of this case lies in the direct use of the patient’s autologous TILs to construct the PDOTS and the effective maintenance of T-cell activity through optimized microfluidic chip technology, thereby potentially providing a more physiologically relevant representation of the *in vivo* immune response process. This advantage is particularly critical when predicting combination immunotherapy regimens (such as anti-angiogenic drugs combined with ICIs in this case), as anti-angiogenic drugs like Lenvatinib have been shown to enhance immunotherapy efficacy by modulating the TME (8). This observed ex vivo sensitivity to the combination regimen may be partly explained by the known immunomodulatory effects of anti-angiogenic agents such as lenvatinib, which can enhance T-cell infiltration and function within the tumor microenvironment, thereby potentially augmenting the efficacy of PD-1 blockade.

However, this study has certain limitations that need to be addressed in future research: First, although the single case provides a preliminary observation of concordance suggesting the model’s potential, the small sample size limits the generalizability of the conclusions and the findings should be viewed as a single-case observation rather than definitive validation. Given the high heterogeneity of liver cancer, prospective studies in larger cohorts are needed to systematically validate the model’s predictive efficacy for HCC patients with different clinicopathological characteristics (e.g., different etiologies, molecular subtypes). Second, the current DST only evaluated the combination of Lenvatinib and Tislelizumab, not covering other approved or investigational targeted drugs (e.g., Donafenib) or other immune combination regimens, which should be included in future testing panels. Furthermore, the limitations of biopsy samples remain an important consideration: small samples may not fully represent spatial tumor heterogeneity, whereas surgical resection specimens typically provide more comprehensive tumor information. Future studies may help to optimize sampling strategies (e.g., considering multi-point biopsies) and integrate multi-region genomic/transcriptomic sequencing with organoid culture to more accurately reflect tumor heterogeneity. In addition, a key technical consideration is whether the observed killing in PDOTS reflects TIL-mediated anti-tumor activity rather than direct drug-induced toxicity, and future studies should incorporate T-cell functional assays or cytokine profiling to address this. Simultaneously, exploring optimized dynamic culture systems to extend the functional activity maintenance time of T cells in the model represents another important direction for future investigation, particularly for evaluating treatment regimens requiring longer assessment periods (e.g., maintenance therapy).

Recent advances in PDOTS-based functional testing platforms across multiple cancer types have highlighted their potential utility for rapid ex vivo therapeutic evaluation while partially preserving native tumor microenvironmental features. In addition to conventional immune checkpoint inhibitors, these systems may also provide a promising platform for evaluating emerging immunotherapeutic strategies, including monoclonal antibodies, bispecific antibodies, and combination immunotherapy regimens. As functional precision oncology continues to evolve, biopsy-derived PDOTS models may offer translational value for individualized treatment stratification and dynamic therapeutic response assessment in advanced cancers.

In conclusion, this case report provides a preliminary clinical observation of concordance between PDOTS-based drug sensitivity testing and clinical response in a patient with advanced HCC, suggesting a potential role for the PDOTS model in guiding personalized combination immunotherapy decision-making. This model may help to overcome the inherent limitations of traditional PDO/PDX models in retaining the tumor immune microenvironment, offering a platform that incorporates autologous TILs and may partially recapitulate the TME for predicting immunotherapy response in an ex vivo setting, though the degree of immune preservation requires further validation. Despite limitations such as small sample size and limited drug testing spectrum, this case provides preliminary, exploratory proof-of-concept evidence for the application value of the PDOTS model in precision therapy for HCC, particularly in the field of combination immunotherapy. With the ongoing standardization and optimization of model construction techniques (e.g., sample processing, culture conditions, DST endpoint interpretation) and the conduct of prospective, large-sample clinical studies, the PDOTS model holds promise to evolve into a tool to assist personalized treatment decisions in clinical practice. It may help identify HCC patient populations most likely to benefit from specific treatment regimens (especially complex immune combination strategies), potentially contributing to improved survival outcomes and quality of life for patients with advanced HCC.

## Data Availability

The original contributions presented in the study are included in the article/Supplementary Material. Further inquiries can be directed to the corresponding authors.
